# Challenges from Tuberculosis Diagnosis to Care in Community-Based Active Case Finding among the Urban Poor in Cambodia: A Mixed-Methods Study

**DOI:** 10.1371/journal.pone.0130179

**Published:** 2015-07-29

**Authors:** Natalie Lorent, Kimcheng Choun, Shelly Malhotra, Pichenda Koeut, Sopheak Thai, Kim Eam Khun, Robert Colebunders, Lut Lynen

**Affiliations:** 1 Infectious Diseases Department, Sihanouk Hospital Center of HOPE, Phnom Penh, Cambodia; 2 Department of Clinical Sciences, Institute of Tropical Medicine, Antwerp, Belgium; 3 Global Alliance for TB Drug Development, New York, New York, United States of America; 4 CENAT, National Tuberculosis and Leprosy Control Programme, Phnom Penh, Cambodia; 5 Epidemiology and Social Medicine Department, University of Antwerp, Antwerp, Belgium; Fundació Institut d’Investigació en Ciències de la Salut Germans Trias i Pujol, Universitat Autònoma de Barcelona, SPAIN

## Abstract

**Background:**

While community-based active case finding (ACF) for tuberculosis (TB) holds promise for increasing early case detection among hard-to-reach populations, limited data exist on the acceptability of active screening. We aimed to identify barriers and explore facilitators on the pathway from diagnosis to care among TB patients and health providers.

**Methods:**

Mixed-methods study. We administered a survey questionnaire to, and performed in-depth interviews with, TB patients identified through ACF from poor urban settlements in Phnom Penh, Cambodia. Additionally, we conducted focus group discussions and in-depth interviews with community and public health providers involved in ACF, respectively.

**Results:**

Acceptance of home TB screening was strong among key stakeholders due to perceived reductions in access barriers and in direct and indirect patient costs. Privacy and stigma were not an issue. To build trust and facilitate communication, the participation of community representatives alongside health workers was preferred. Most health providers saw ACF as complementary to existing TB services; however, additional workload as a result of ACF was perceived as straining operating capacity at public sector sites. Proximity to a health facility and disease severity were the strongest determinants of prompt care-seeking. The main reasons reported for delays in treatment-seeking were non-acceptance of diagnosis, high indirect costs related to lost income/productivity and transportation expenses, and anticipated side-effects from TB drugs.

**Conclusions:**

TB patients and health providers considered home-based ACF complementary to facility-based TB screening. Strong engagement with community representatives was believed critical in gaining access to high risk communities. The main barriers to prompt treatment uptake in ACF were refusal of diagnosis, high indirect costs, and anticipated treatment side-effects. A patient-centred approach and community involvement were essential in mitigating barriers to care in marginalised communities.

## Introduction

Early detection and treatment of tuberculosis (TB) are critical to containing the spread of the disease.[1] Active case finding (ACF) is a novel approach to TB screening and a promising tool for increasing early case detection among marginalised populations.[2–4] As opposed to passive case-finding it involves systematically searching for TB in individuals who would not spontaneously present for care. Prompt linkage of diagnosed TB patients to treatment is crucial to ACF’s success, as failure to do so may result in disease progression and continued transmission within the community.[5] In passive case-finding settings, delayed or failed linkage to care has been reported in 8–38% of TB cases.[6–9] Long turn-around times for results, negative perceptions of national tuberculosis programmes’ working procedures,[10] lack of knowledge about TB,[10,11] fear, stigma,[12] access barriers,[13] and financial constraints[11,14] are some of the documented causes for delays in treatment initiation across national settings. In theory, by bringing screening and/or diagnostic services closer to patients, ACF could overcome many barriers related to late presentation among TB patients, especially in settings with limited access to or affordability of care; however, there remains limited understanding of optimal implementation and integration of ACF services both at patient and health system level in diverse socio-economic, cultural, and national contexts.

Our own door-to-door TB screening programme in Cambodia contributed substantially to case notifications in the capital, but initially up to 20% of actively diagnosed TB patients delayed treatment initiation, despite substantial efforts to facilitate care.[3] In a separate household contact screening programme in Cambodia, the initial lost to follow-up rate was reportedly 5%,[15] whereas in a mobile TB/HIV-screening programme in South-Africa, it was 16%.[16] Reasons for failure to initiate treatment have not yet been fully explored. Understanding real and perceived barriers faced by TB patients and other key stakeholders will likely improve the effectiveness and affect impact of ACF.

This mixed-methods study evaluates perspectives of patients and health care providers participating in ACF and explores reasons for delayed or failed linkage to care. At a time when innovative approaches for case detection among high risk populations are being scaled up and codified by agencies such as the World Health Organisation, findings from this study can offer practical guidance for further policy development both at the national and global levels.

## Methods

### Ethical considerations

Ethical approval for the study was granted by the National Ethics Committee for Health Research of Cambodia, the Ethics Review Committee of the Western Pacific Regional Office of the World Health Organisation, the Institutional Review Board of the Institute of Tropical Medicine, and the Ethics Committee of the University Hospital, in Antwerp (Belgium). All participants provided written or thumb-printed informed consent.

### Study site and setting

TB prevalence in Cambodia remains among the highest in the world,[1] with 831 bacteriologically confirmed cases per 100,000 population.[17] Detection (mainly on symptomatic patients voluntarily presenting to health facilities) and treatment of TB are provided free of charge by the national tuberculosis programme. In line with the World Health Organisation’s recommendations, directly observed treatment short-course (DOTS) is offered at health centres and district hospitals. Average distance to a health facility in Cambodia is 4,8 km,[18] and clinic operating hours are often limited to early mornings. In the capital, community-DOTS (c-DOTS) is implemented by only a few institutions, including the Sihanouk Hospital Center of HOPE (SHCH), a large tertiary care non-govermental hospital in Phnom Penh with satellite community-based programmes throughout greater Phnom Penh.

Alongside the national tuberculosis programme, and with support from the Stop TB Partnership/TB REACH Initiative, the Sihanouk Hospital Center of HOPE launched a large scale ACF project in Phnom Penh in 2012. Over a two-year period, SHCH conducted door-to-door screening for TB in vulnerable and poor communities of Phnom Penh, in collaboration with 14 health centres and 3 district referral hospitals, as previously described.[3]

### Study Design

We performed a mixed-methods study combining quantitative and qualitative data.

All interviews were held at local health facilities between 19 September and 11 October 2013. Transportation costs were reimbursed.

To ensure objectivity and avoid response bias, interviews were performed by two experienced, independent, native Khmer interviewers. The interviewers were monitored and supported throughout the study by KC.

#### Quantitative component

We conducted a cross-sectional survey of adults (15 years or older) diagnosed with TB through SHCH’s ACF project.[3] To avoid recall bias, we initially recruited only individuals who had been diagnosed within the previous 6 months. Patients who initiated TB treatment without delay were selected using probability proportionate to size random sampling per operational health district. In addition, all those who delayed or failed to initiate treatment were purposively sampled given our interest in capturing the perspectives of patients facing difficulties linking to care. Because of persistent enrolment challenges with this priority sub-group, we eventually broadened our inclusion criteria to capture patients diagnosed within the previous 9 months.

Given the low level of literacy in target communities, interviewers verbally administered a structured questionnaire that covered both demographics and topics such as the acceptability of door-to-door TB screening, previous health seeking behaviour, and views on the ACF process. Standard descriptive statistics were used to characterize the study population. Odds ratios and 95% confidence intervals (CI) were used to compare dichotomised patient characteristics, and parameters on health seeking behaviour. All analyses were performed using STATA software version 10.0 (College Station, Texas, USA).

#### Qualitative study

We conducted in-depth interviews with patients diagnosed through the project to gain insight into facilitators and barriers from diagnosis to TB treatment completion within ACF approaches. We also held focus group discussions with participating community TB workers (staff specifically recruited and trained for the project) and village health volunteers (lay volunteers from the existing community health network) to obtain their perspectives. Participants were purposively sampled to ensure gender balance, representation across experience levels, and geographic diversity. We convened six focus group discussions in total: four with village health volunteers (each consisting of five to six participants) and two with TB workers (each consisting of nine individuals) involved in ACF.

Because the ACF project was embedded in national TB programme services, we also interviewed public sector providers. Seventeen public sector health centre staff (one from each facility in the catchment area), three laboratory technicians (one from each district lab), and two TB supervisors (one from each of the two main operational districts in the study) participated in in-depth interviews. The median TB experience of workers was 10 years.

Interviewers used open-ended discussion guides, which covered topics such as screening preferences, health seeking behaviour, pathways to treatment, and challenges encountered. The interview guide for health providers addressed topics such as project design, interactions between ACF stakeholders, achievements, and challenges.

All sessions were held in Khmer, the local language, and with the permission of participants, were audio-recorded. They were transcribed verbatim and translated into English by the interviewers, assisted by KC and PK.

The data were coded manually. An initial coding framework based on commonly reported facilitators and barriers was developed by KC, PK and NL. KC, NL, and SM independently reread the transcripts in English to identify keywords and emerging themes. They subsequently organised and coded the data. Results were compared and discrepancies discussed.

### Definitions

Smear-positivity was defined as at least one smear containing at least one acid-fast bacillus.[19] All patients with a positive smear, or *M*. *tuberculosis* detected by Xpert or isolated by culture were considered to have bacteriologically-proven TB.[20] TB treatment was initiated based on the first available positive result or clinical indication in the absence of bacteriological evidence (clinical TB). Time to treatment initiation for bacteriologically-confirmed TB was defined as the interval between the first positive result and the start of treatment. Delayed linkage to treatment was defined as more than one week delay between diagnosis and TB treatment initiation. When a patient diagnosed with TB failed to initiate treatment, he/she was considered a pre-treatment loss to follow-up.[20,20,21]

Community workers include TB workers-paramedical staff recruited by the project and trained for the purpose- and village health volunteers—lay workers from the existing national community health network.

## Results

**Fig 1:**
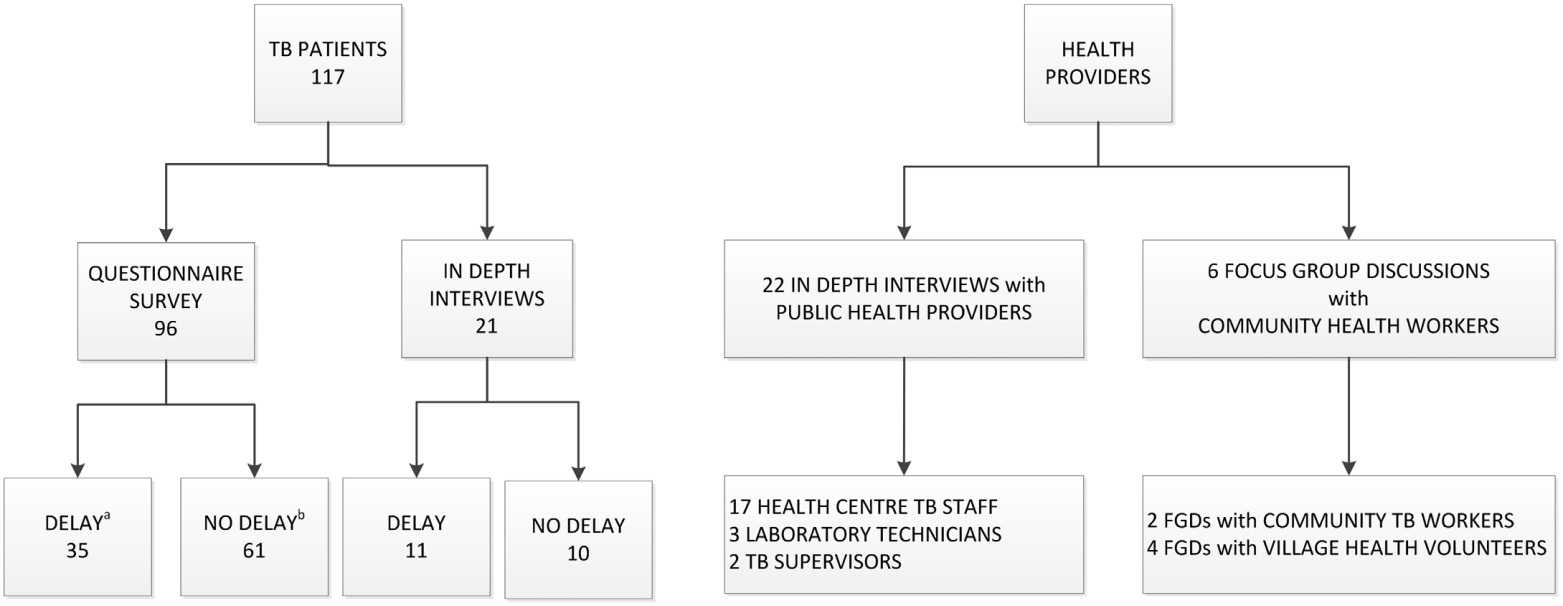
Key participants in the mixed-methods study on perspectives of community-based active case finding in Cambodia. ^a^ Enrolment through purposeful sampling; ^b^ enrolment through probability proportionate to size sampling. TB: tuberculosis, FGD: focus group discussions.

In total, 117 of the 185 TB patients approached participated in the study; 68 could not be contacted or refused participation. Of 80 TB patients who delayed/failed treatment uptake, 46 (57%) agreed to participate in the study (Fig 1).

### Quantitative survey

Table 1 shows the baseline characteristics of all 96 TB patients enrolled in the survey: 61 (63%) initiated treatment without delay and 35 (36%) experienced delays. TB patients aged 60 or more, those who are not or no longer married and those living outside a five kilometre radius of the health centre had a significantly higher risk of delaying or failing treatment initiation; whereas a positive smear result had a protective effect on treatment delay.

**Table 1:** Baseline characteristics of the 35 tuberculosis patients participating in the survey, who delayed or failed tuberculosis treatment initiation and the 61 who initiated without delay.

Patient characteristics	Delay (n = 35)	No delay (n = 61)	Odds ratio*
Age in years, median (IQR)	53 (35–65)	47 (33–50)	
age <60	18 (51,4)	46 (75,4)	1
age > = 60	17 (48,6)	15 (24,6)	2,90 (1,20–7,00)
Gender, n (%)			
Male	21 (60,0)	37 (60,7)	1
Female	14 (40,0)	25 (39,3)	0,97 (0,42–2,27)
Marital status, n (%)			
Married	29 (82,9)	38 (62,3)	1
Single/widowed/divorced	6 (17,1)	23 (37,7)	2,93 (1,05–8,11)
Education, n (%)			
Primary or higher schooling	29 (82,9)	50 (82,0)	1
No schooling	6 (17,1)	11 (18,0)	0,96 (0,36–2,56)
Occupation, n (%)			
Regular income	14 (40,0)	15 (24,6)	1
No regular income	21 (60,0)	46 (75,4)	1,72 (0,73–4,05)
Distance to nearest health facility, n (%)			
1–5 km	18 (51,4)	49 (80,3)	1
>5 km	17 (48,6)	12 (19,7)	3,86 (1,54–9,62)
Smear results			
Negative	25 (71,5)	30 (49,3)	1
Positive (1+ or more)	10 (28,5)	31 (50,7)	0,39 (0,16–0,94)
Time from diagnosis to treatment, median (IQR)	12 (9–17)	2 (1–3)	

* Unadjusted odds ratio

All but one participant found home screening for TB more convenient than facility-based screening; however, 26 (27%) perceived home visits as intrusive and eight (8%) cited privacy concerns. The majority of participants (91%; n = 87) preferred having a village health volunteer accompany a community TB worker, rather than having the latter approach community members on their own. Sputum collection at home was deemed convenient, but 16 (17%) found it embarrassing.

Table 2 explains the health seeking behaviour prior to home visits. Approximately 70% of patients had experienced symptoms for more than two weeks at the time of screening. The majority of patients -79% and 64% of those delaying and initiating treatment, respectively—had already sought care at a health facility. The most common reason for not seeking care while being symptomatic was that they did not feel sick (enough).

While only 35/96 survey participants delayed or failed to initiate TB treatment, 89/96 (83%) reported difficulties starting and/or continuing treatment.

**Table 2:** Quantitive survey data on health seeking behaviour prior to home visits for the 35 tuberculosis patients who delayed or failed tuberculosis treatment initiation and the 61 who initiated without delay.

	Delay (n = 35)	No delay (n = 61)	Odds ratio
Duration of TB symptoms^a^, n (%)			
Less than 2 weeks	12 (33)	18 (30)	1,30 (0,53–3,18)
2 weeks of more	22 (67)	43 (70)	1
Aware of the need to be screened before ACF^b^, n (%)			
Yes	11 (33)	29 (48)	1
No	22 (67)	32 (52)	0,55 (0,23–1,33)
Consulted a health facility before ACF visited home, n (%)			
Yes	26 (79)	39 (64)	1
Health facility visited, n (%)			
Pharmacy	15 (58)	15 (38)	
Private clinic	8 (31)	7 (18)	
Traditional healer	0 (0)	1 (2)	
Public/NGO-run facility	3 (11)	16 (41)	
No	7 (21)	22 (36)	2,10 (0,78–5,61)
Reason for not seeking care while symptomatic, n (%)			
Did not know where to go	0 (0)	1 (5)	
Too busy with work	0 (0)	4 (19)	
No money to travel	1 (14)	3 (14)	
Not sick (enough)	6 (86)	13 (62)	

^a^ 1 missing symptom duration among the delayers

^b^ 2 missing answers among the delayers. ACF: active case finding, NGO: non-governmental health facility, TB: tuberculosis

### Qualitative study

#### TB patients

In all, we interviewed 21 TB patients: 11 who delayed or failed linking to treatment and 10 who successfully initiated treatment. We purposefully sampled patients by convenience.

Overall participants were very supportive of home-based TB screening. By bringing TB services closer to the community, participants reported that ACF had removed barriers of access and cost. This was particularly appreciated by the elderly and severely ill, whose physical conditions pre-empted them from traveling to the health centre for screening and treatment:

*“I thought that when I was free I would go to screen for TB at the health centre… When they came to screen for tuberculosis at my home*, *it was like my second chance for survival*. *It is good*. *In my life I never met a doctor who came to visit my house*.*” (male TB patient*, *age 55)*

While TB treatment was provided free of charge through the national tuberculosis programme, most TB patients (85%) reported indirect costs hindering prompt uptake of and adherence to treatment. Long distances to health facilities and transportation challenges aggravated this situation. For those working in the informal sector, where wages are low and work is not steady, the requirement to attend daily facility-based DOTS placed them at risk of loss of livelihood.

*“To get to the health centre*, *I lose time from my work*. *I lose a half day of work to go to get drugs*. *And*, *I must pay for a motodop (motorcycle taxi)*.*” (female TB patient*, *age 20)*

*“I had to wait so long at the hospital for treatment until my turn… I didn't pay money for the drugs but I paid money for petrol to go there*. *When I went to hospital I had to leave my children with the neighbours*.*” (male TB patient*, *age 29)*

Of the 11 patients participating in in-depth interviews who delayed treatment initiation, six attributed it to their doubting the diagnosis, feeling otherwise healthy:

*“At that time I didn't believe that I had TB*. *I wondered and was surprised because my health was still strong*. *I felt normal*.*” (male TB patient*, *age 37)*

Some reported a lack of clarity about the severity of their illness:

*“They asked to take my sputum*. *I was wondering what are they taking it for*? *They asked for sputum but did not tell me what would happen to me*. *Then they told me that I was ok*. *They did not tell me whether I had small or big TB*. *They did not speak out*.*” (male TB patient*, *age 80)*

In line with quantitative findings, anticipated and actual side-effects from anti-tuberculosis drugs were a major concern for patients, influencing their decision to initiate and complete treatment, respectively. Many patients feared side-effects based on reports from friends, neighbours, and other patients. Some experienced debilitating side-effects early in the course of treatment, resulting in lost productivity. Concerned about further loss of income or debilitation, they subsequently discontinued treatment.

*“When I took the drugs I felt pain in my extremities*. *My arms and feet felt like crabs’ legs being broken until the drug was stopped*. *Then I felt better*.*” (male TB patient*, *age 52)*

*“I didn't take my TB medication because I have a lot of diseases such as gastritis*, *hypertension*, *and heart problems and I (already) take so many drugs to treat those*. *I also heard other patients talking about the side effects of the drugs so I was afraid I couldn't take it*. *The TB worker came a few times to my home to convince me but my health was so weak so I decided not to take TB medication until now*.*” (male TB patient*, *age 59)*

One of the most powerful contributors to patient compliance—both among those initially non-adherent and those with successful treatment uptake—was familial pressure; inversely, a lack of family support presented challenges in terms of adherence.

*“I never drop my medicine*. *You can check the pills of medicine*. *If I drop my medicine*, *my daughter will be upset with me*.*” (female TB patient*, *age 43)*

*“Yes*, *my wife is careful*. *She pressures me because she’s afraid that if I don't take the drugs regularly*, *I will die and no one will take care of my children*. *They are small*.*” (male TB patient*, *age 70)*

*“I faced difficulties*. *All my children went to work*. *No one was available to accompany me*. *I had difficulty walking because of my poor vision*.*” (female TB patient*, *age 77)*

When asked how treatment uptake and adherence in ACF could be improved, participants recommended more comprehensive patient education by health workers about TB treatment duration, side-effects, and adherence; ongoing encouragement; and a more flexible treatment delivery approach.

*“Encouragement (by health workers) is not usual for the poor*. *But also for us poor*, *a respectful and courteous approach (by health workers) can do miracles*.*” (female TB patient*, *age 40)*

*“If they have difficulty with taking drugs*, *we should explain to him/her that the drugs make you uncomfortable for around half to one month only*. *Please be patient and complete it for 6 months*.*” (male TB patients*, *age 37)*

*“They should provide drugs at home one time a week or a month because at the village*, *a lot of poor people are waiting for help because they are poor*. *They lack transportation to reach the health centre*.*” (male TB patient*, *age 37)*

Participants also emphasised the need for health education, particularly on TB. They recommended expanded community involvement in this process, including stronger peer support networks:

*“I am a former TB patient*. *Now I’ve been cured from TB*. *I want other patients who have not yet received treatment to get treatment from the doctor and get cured like me*.*” (female TB patient*, *age 43)*

*“Have volunteers go down with the doctor*. *It helps our community to develop on the health side*, *because volunteers in the village know their people*.*” (male TB patient*, *age 58)*

#### Health providers

Community TB workers and village health volunteers considered themselves critical and complementary links between marginalised, at risk communities and the public health system. Nevertheless, community workers identified several challenges implementing ACF. In target communities, workers were sometimes shouted at, ignored, or harassed and had to endure extreme weather conditions.

*“Yes*, *the first day I considered returning home… People were busy playing cards*, *saying “yes*, *so why did you come here*?*” It was absolutely meaningless*.*” (female village health volunteers)*

*“We walk through water*, *mud and then when we go there*, *we have to be patient*.*” (female TB worker)*

In several communities, such as factory areas with high numbers of migrant labourers, community health workers visited on weekends or after working hours in order to find patients at home.

*“Normally*, *the people in Steung Meanchey (factory area in Phnom Penh) are migrant people*, *working for their daily subsistence*. *When our team misses them*, *we have to adapt to their schedules*. *Sometimes we wait from 4pm until 7pm to meet them*.*” (female TB worker)*

*“Overall*, *construction workers never have free time during the day*, *so we spare our time to meet with them on Sunday at 4 or 5 pm*, *outside of work hours we go visit their house” (male TB worker)*

Limited understanding, frequent relocation, and pressing needs related to housing insecurity, food shortages, addiction, and violence presented challenges in terms of recruiting and retaining patients. Given the fundamental subsistence struggles faced by target populations, convincing them to start treatment was arduous and time consuming:

*“The places that we work*, *the family situation is bad*: *there is poverty*, *poor knowledge*, *too many children*. *When we ask them*, *they said "Oh*, *I don't care*. *I can't even find rice to fill a pot*.*" (female TB worker)*

*“He didn't know he had tuberculosis*. *I mean that sometimes he had a cold or a chronic cough but he had no money to pay for transportation to get to the health facility… so he decided to sleep until he died*! *Yes*, *he didn't come because he didn't have money*.*” (male TB worker)*

Tracing TB patients lost to follow-up or for routine treatment monitoring was also a major concern for health workers, particularly in informal settlements with large concentrations of internal migrants and individuals with no fixed abode.

*“It's difficult to find the patients also because the majority of the people who live in the villages covered by my health centre are migrants from others provinces or others places*, *and don't have permanent homes*. *That is why we have difficulty managing and difficulty finding them*.*” (male nurse at health centre)*

Consistent with patient responses, TB workers expressed concern about ensuring patient adherence to DOTS, given irregular operating hours at health centres and challenges faced in traveling to facilities for daily observed therapy. They saw the requirements of facility-based DOTS as significantly contributing to treatment delay and non-adherence:

*“It was difficult for some to get TB treatment at the health centre because the doctor was not always there*. *Also*, *some (doctors) were flexible*, *but not all*. *One was very strict*. *He did not allow the patient to take the drugs at home*. *He wanted the patient to come to the health centre every morning*. *So he (the patient) had no choice but to defer treatment*!*” (male TB worker)*

To increase treatment initiation and adherence among patients identified through ACF, community workers and providers became creative in tailoring treatment delivery to patients’ needs and preferences. Strategies included allowing more liberal indications for c-DOTS and promoting more flexible facility-based DOTS.

Consistent with quantitative findings, several health providers attributed delayed treatment initiation and loss to follow-up to the higher prevalence of less advanced disease among patients identified through active case detection. They found those feeling asymptomatic and otherwise strong to be more reluctant to accept their diagnosis, and less likely to initiate and adhere to treatment. The debilitating side effects of anti-tuberculosis medicines compounded these challenges.

*“Because of early detection*, *we called them to offer advice*. *Sometimes they argued and denied (their condition)*. *They would say they are healthy*. *Why did we think they had the disease*? *They didn't trust us because they were still feeling strong*.*” (male TB nurse at health centre)*

*“When we go to the patients’ house*, *we beg them to get treatment*. *That is right*, *we beg them to get treatment*, *but when they get treatment*, *they get drug side effects that makes them stop taking the drugs*. *That is the main reason that they gave up” (male TB staff at health centre)*

TB workers saw village health volunteers as important liaisons and informants in navigating access to target communities. Unfortunately, village health volunteers’ subsistence needs sometimes undermined their ability to tend to ACF responsibilities. As such, health workers stressed the importance of adequately compensating community volunteers for time committed to ACF:

*“We should have more volunteers help us than now but they require a higher incentive to encourage them to work actively*. *For example when we asked them to help us sometimes they said they had no time*. *If we had more money*, *they said they would have time for us*.*” (male TB workers)*

While village health volunteers’ familiarity and knowledge of the community proved essential in facilitating initial access to ACF participants, at times, it inversely tempered their perceived legitimacy among community members, who were aware of their lack of medical training. Village health volunteers saw affiliation with TB workers and other trained health care providers as important in gaining respect among peers in the community:

*“When we said that we came from HOPE centre (SHCH)*, *people knew that we lived in the same village as them and never attended medical college*, *so they did not have confidence in us*. *Then*, *next time when we went with the TB workers*. *People seemed to listen*.*” (female village health volunteer)*

Public health providers were motivated by the increased early case detection and expedited sequence from screening to treatment thanks to the ACF intervention. TB workers were proud to see how their community visits had increased awareness, improved health seeking behaviour among TB patients and strengthened the competence of village health volunteers in target communities.

*“Now the workload is higher; before we had a lot of free time*. *Now we are busy with the patients but we are happy*.*” (medical doctor at referral hospital)*

*“The project went down to the community level*. *That’s why currently people understand more… around 90–95%*. *Even when they just have a little cough they come to the health centre because HOPE (SHCH) went down to provide education and training on the signs of TB*. *Previously*, *patients didn't understand*.*” (male nurse at health centre)*

Additional financial support for staff time and for program costs related to the scale-up in patient numbers were seen as important factors in sustaining public sector engagement in ACF.

## Discussion

There has been renewed interest of late in reaching the 3 million individuals with active TB infection each year who currently fall between the cracks in TB control efforts. [22] Innovative programmes such as the Stop TB Partnership’s TB REACH initiative have been at the forefront of strategies to identify hard-to-reach populations through the scale-up of ACF for TB. While a body of literature has emerged examining the effectiveness of various ACF approaches, the acceptability of ACF has, to date, received scant attention. Using participation rates as a proxy for acceptability, support for ACF is generally presumed to be high;[23] however, participation rates alone are of limited value in understanding the optimal applications of ACF and informing policy change. Our study is one of the first to systematically explore the perspectives of patients and health providers engaged in community-based ACF. Findings suggest a high level of acceptability for home-based ACF across key stakeholders, including TB patients, village health volunteers, community TB workers, and public sector providers.

For ACF to have an impact on TB control, early case detection must be followed by successful treatment initiation and completion. In our target population, indirect costs posed significant barriers to treatment success. Consistent with previous studies, our findings suggest that in many cases, “free tuberculosis diagnosis and treatment are not enough” [24] given the multitude of costs[25] that TB patients face related to transportation[13] and lost income[26], productivity[10], and time[6].

Other leading barriers to treatment uptake identified in the study were non-acceptance of diagnosis and fear of drug side-effects. By proactively seeking out patients at risk of TB, rather than passively capturing patients seeking treatment of their own volition, ACF has the benefit of reaching individuals in earlier stages of disease. An indirect consequence of early detection, however, is that individuals may not self-identify as ill and may be reluctant to accept their diagnosis.[5,27] Since, in general, ACF patients initiate treatment from a healthier baseline, the real and/or perceived discomfort from anti-TB drugs may dwarf the perceived benefits of treatment initiation and adherence. These factors contribute to rates of initial loss to follow-up in ACF as high as 25% [4,16] potentially undermining the gains made through increased case detection.

In Cambodia, as in several African countries,[28–32] involvement of community volunteers and family members in TB treatment has proven an effective strategy for cost reduction both at the patient and health sector levels, while ensuring good treatment outcomes.[33] Our study affirms and further clarifies these findings. By reducing access barriers and shifting both direct and indirect costs away from patients, community-based ACF approaches make important contributions to TB screening and diagnosis among resource limited populations.[3,4] Treatment models that were responsive to patients preferences and needs, by leveraging community involvement and bringing TB services closer to the patient, the initial loss to follow-up rate in our programme was reduced from 20% (during the first few months of the project) to 5%, by program’s end.[3]

While village health volunteers played an important value-added role as facilitators and liaisons, lack of knowledge, high workload, and low remuneration limited their ability to optimally function. As suggested by Gilson et al.[34] in the late 1980’s, community health workers’ services need to be integrated into the health system to maximize their potential and ensure sustainability; however, providing this extension of service to marginalised communities is neither cheap nor easy, and requires careful assessment of additional costs versus benefits. As such, the scalability and cost-effectiveness of community and home screening for TB requires further evaluation.[5,35,36]

### Limitations

The purposive sampling of patients who delayed/failed treatment (as opposed to probability sampling for those without delay) in the quantitative part made comparison of risk distribution difficult. We choose purposive (total) sampling of TB patients who delayed or failed to link to treatment because studying barriers to care was one of our main objectives. Recruiting them for the study proved challenging: of all patients who experienced difficulties linking to care, we were able to track only 57%. It may have influenced the representativeness of our quantitative data; however, through triangulation with qualitative data, we were able to further validate quantitative findings.

To encourage objectivity and respondent openness, we used independent interviewers for the study. A drawback of this approach was that by using individuals with limited knowledge of the project, we may have missed opportunities to delve deeper into areas of substantive interest.

Another challenge related to the translation of findings from Khmer to English, which could have contributed to distortion of meaning. This was abetted through use of translators who are fluent in both languages, and through regular discussions between interviewers and researchers to clarify intended meaning.

Finally, given the specific national context and setting of our study, findings may not be widely generalizable; however parallels are likely in similar low-resource, high burden urban contexts.

## Conclusion

As one of the first systematic explorations of local perspectives and context-specific barriers from diagnosis to care in ACF interventions, this study offers important insight that can inform effective programme implementation in Cambodia and similar contexts.

Findings confirm the high acceptability of ACF among key participants, and its potential for additional case detection yield among hard-to-reach populations. Nevertheless, this study highlights refusal of diagnosis, high indirect costs, and anticipated/real treatment side effects as leading barriers to prompt treatment uptake and adherence in ACF. A patient-centred approach was essential in mitigating barriers to successful TB treatment initiation and completion in ACF approaches. Addressing issues related to training, task allocation and financial compensation for health providers, in particular at the community level, is also critical to ensuring quality service and sustained commitment among stakeholders.

## Supporting Information

S1 File Coding interviews with health staff. (XLS)

S2 File Coding interviews with community tuberculosis workers. (XLS)

S3 File Coding interviews with tuberculosis patients. (XLS)
